# Retraction: Estrogen deprivation aggravates cardiac hypertrophy in nonobese Type 2 diabetic Goto–Kakizaki (GK) rats

**DOI:** 10.1042/BSR20170886_RET

**Published:** 2026-08-25

**Authors:** Nattayaporn Apaijai, Narattaphol Charoenphandhu, Jitjiroj Ittichaichareon, Panan Suntornsaratoon, Nateetip Krishnamra, Ratchaneevan Aeimlapa, Siriporn C. Chattipakorn, Nipon Chattipakorn

**Affiliations:** 1 Cardiac Electrophysiology Research and Training Center, Faculty of Medicine, Chiang Mai University, Chiang Mai 50200, Thailand; 2 Cardiac Electrophysiology Unit, Department of Physiology, Faculty of Medicine, Chiang Mai University, Chiang Mai 50200, Thailand; 3 Center of Excellence in Cardiac Electrophysiology Research, Chiang Mai University, Chiang Mai 50200, Thailand; 4 Center of Calcium and Bone Research (COCAB), Faculty of Science, Mahidol University, Bangkok 10400, Thailand; 5 Department of Physiology, Faculty of Science, Mahidol University, Bangkok 10400, Thailand; 6 Department of Oral Biology and Diagnostic Sciences, Faculty of Dentistry, Chiang Mai University, Chiang Mai 50200, Thailand

**Keywords:** cardiac hypertrophy, diabetes, mitochondrial dysfunction, oxidative stress

This article is being retracted from *Bioscience Reports* at the request of the authors following receipt of a notification from a reader, alerting the Editorial Office to concerns regarding the representative Western blot images presented in Figure 3. Specifically, the Figure 3B Bcl-2 bands for the WTO and GKS groups appear similar to the Figure 3D β-actin bands for the GKS and GKO groups, and a potential splice site was noted between the p-p38 bands of the WTO and GKS groups in Figure 3C.

The authors conducted an internal investigation, during which the first author, who performed the Western blot experiments, explained that a placeholder image was inadvertently not replaced with the correct representative β-actin blot during figure preparation. The authors made every effort to recover the original archived data; however, given the age of the study, many of the original Western blot image files, including the uncropped scans, are no longer recoverable. Consequently, the authors are unable to determine with certainty how the error in Figure 3C occurred.

To uphold the principles of research integrity and maintain the trust of the scientific community, the authors have decided to voluntarily retract the article. They sincerely apologize for these errors and for any inconvenience they may have caused. The Editor-in-Chief and Editorial Board agree with the retraction.

